# Intra- and Inter-Day Reliability of the NIRS Portamon Device after Three Induced Muscle Ischemias

**DOI:** 10.3390/s22145165

**Published:** 2022-07-10

**Authors:** Julien Desanlis, Dan Gordon, Camille Calveyrac, François Cottin, Marie Gernigon

**Affiliations:** 1 CIAMS, Université Paris-Saclay, CEDEX, 91405 Orsay, France; calveyraccamille@gmail.com (C.C.); francois.cottin@universite-paris-saclay.fr (F.C.); marie.gernigon@universite-paris-saclay.fr (M.G.); 2 CIAMS, Université d’Orléans, 45067 Orléans, France; 3 Anglia Ruskin University, Cambridge CB1 1PT, UK; dan.gordon@aru.ac.uk

**Keywords:** test–retest, arterial occlusion, blood flow restriction, ischemic preconditioning, hyperemia

## Abstract

(1) Background: Near-infrared spectroscopy (NIRS) is an innovative and non-invasive technology used to investigate muscular oxygenation. The aim of this study is to assess the within- and between-session reliability of the NIRS Portamon (Artinis, Elst, Netherlands) device following three sets of induced muscle ischemia. (2) Methods: Depending on the experimental group (G1, G2 or G3), a cuff was inflated three times on the left upper arm to 50 mmHg (G1), systolic blood pressure (SBP) + 50 mmHg (G2) or 250 mmHg (G3). Maximum, minimum and reoxygenation rate values were assessed after each occlusion phase, using a Portamon device placed on the left brachioradialis. Reliability was assessed with intraclass correlation coefficient (ICC) value and ICC 95% confidence interval (CI-95%), coefficient of variation (CV) and standard error of measurement (SEM) (3) Results: Our results showed a good to excellent reliability for maximums and minimums within-session. However, the reoxygenation rate within sessions as well as measurements between sessions cannot predominantly show good reliability. (4) Conclusions: Multiple measurements of maximums and minimums within a single session appeared to be reliable which shows that only one measurement is necessary to assess these parameters. However, it is necessary to be cautious with a comparison of maximum, minimum and reoxygenation rate values between sessions.

## 1. Introduction

Near-infrared spectroscopy (NIRS) is an innovative and non-invasive technology used to investigate muscular oxygenation through several indicators. By using near-infrared wavelengths (~700–900 nm), the light is able to penetrate biological tissues where the main absorbing chromophores in skeletal muscle are hemoglobin (Hb), myoglobin (Mb), and to a lesser extent, cytochrome oxidase (cytox) [1,2]. Thus, NIRS technology provides information on four variables, depending on the device used: oxy[Hb + Mb], deoxy[Hb + Mb], total[Hb + Mb] (i.e., the sum of oxy- and deoxy[Hb + Mb]) and tissue saturation [1]. The concept of recording muscle oxygenation light dates back to 1937 [3], and human skeletal oxygenation measurements to the end of the 1980s [4].

Due to their moderate cost, convenient small size and wireless connectivity, NIRS devices have been widely used at rest and during exercise [5,6,7,8,9]. Moreover, NIRS oximeters offer the advantage of acceptable signal-to-noise ratios, even during dynamic exercise [8]. Indeed, NIRS provides robust information on skeletal muscle oxidative capacity. NIRS-derived parameters such as the muscle reoxygenation rate after exercise [5,10,11], the muscular consumption of O_2_ (m$\dot{V}O_{2}$) [12], as well as the size of the post-occlusive reactive hyperemia (PORH) [13] may be used to assess performance, vascular reactivity and training status. PORH is characterized by an increase in blood flow following an arterial occlusion, representing the significance of limb reperfusion after ischemia [14,15]. This physiological reaction seems to be caused by the shear stress induced by arterial occlusion acting at the endothelial cell surface and metabolites released by the endothelium [15,16]. PORH is primarily used as a non-invasive method to assess peripherical microvascular function and cardiovascular morbidity and mortality [14].

The use and the interest of NIRS in monitoring skeletal muscle oxygenation have been well documented in the literature through multiple reviews as well as the evolution of the available technology and oximeters [1,7,8,17]. By using NIRS-derived reoxygenation rate parameters, it is possible to differentiate between trained and untrained participants [8], as well as healthy participants and diseased patients [18]. Thus, it is fundamental to assess the reliability of these measurements.

NIRS studies showed good to excellent reliability during static [19,20] and dynamic exercise [21,22,23], as well as at rest [22,24] following arterial occlusions over the systolic blood pressure (SBP). Reliability has been studied for intra-day measurements, between successive measurements of a single session, and for inter-day measurements, by assessing the reliability between measurements of separate sessions. Indeed, excellent reliability has been shown for both intra-day and inter-day (intraclass correlation coefficients (ICC) of 0.92 and 0.94, respectively) [24]. However, in most studies, the 95% confidence interval (CI-95%) was not reported, potentially leading to confusion by changing the interpretation of the level of reliability [25]. The reliability of maximum and minimum values of NIRS variables reached after arterial occlusion are rarely investigated, yet they provide information on the amplitude of the deoxygenation during occlusion and reoxygenation after cuff release [2].

Previous studies have investigated the muscle tissue oxygenation responses to different levels of occlusion pressure [26] as well as the effect of occlusion duration on muscular oxygenation [27]. However, the reliability of multiple NIRS-derived parameters between trials of a single session and between sessions, with different levels of occlusion pressure, has never been investigated to our knowledge. Thus, the present study aims to investigate the intra-day and inter-day reliability of NIRS-derived parameters, as well as the associated maximum and minimum responses, after three blood flow occlusions with different levels of pressure whilst in the rested state.

## 2. Materials and Methods

### 2.1. Participants

Following local institutional ethical approval (CER-Paris-Saclay-2020-006) and having provided written informed consent, 53 young males (mean ± SD: 23 ± 3 years, 75 ± 12 kg, 178 ± 7 cm, 13.8 ± 4.1% body fat, 7.8 ± 5.7 h of physical activity per week) volunteered to participate. The participants’ characteristics, as well as the environmental conditions, are reported in Table 1 and Table 2. Participants who reported diabetes, hypertension, previous cardiovascular events, vigorous physical activity, alcohol intake 24 h before the session, caffeine, or food intake two hours before the session were excluded from the study.

Participants visited the laboratory twice within a 24 h to 72 h period. During the first visit, anthropometric measurements were recorded (stature, body mass, and adipose tissue thickness), while limb lengths were measured to ascertain NIRS device placement.

### 2.2. Study Design

**Figure 1:**
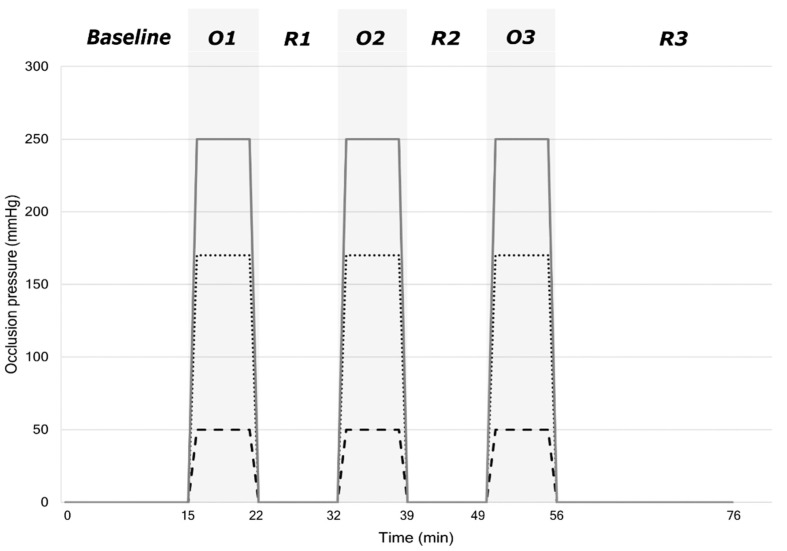
Participants were lying on a medical couch for the duration of the session in the dark and asked to avoid any movement which could disturb the signal. After a 15 min baseline period, the cuff placed on the left arm was inflated to induce three occlusion phases (O1, O2, O3) each of seven minutes. Each occlusion phase was followed by a reperfusion period (R1 = 10 min, R2 = 10 min, R3 = 20 min).

Using an experimental design, participants were randomly assigned to one of three groups with different occlusion pressures: 50 mmHg (G1), SBP + 50 mmHg (G2) and 250 mmHg (G3) [28,29]. The protocol is described in Figure 1 and consists of a baseline period followed by 3 × 7 min occlusion phases interspersed with three reperfusion phases where the cuff was deflated.

### 2.3. Measurements

#### 2.3.1. Skinfolds

Triplicate measurements of skinfold thickness were attained from four-sites (nondominant subscapular, bicipital, tricipital, and suprailiac) using a Harpenden Skinfold Caliper (Baty International, Wantage, United Kingdom). Skinfold thickness was calculated as the sum of the average values of the four sites. Fat percentage was determined with the correlation table provided by the manufacturer. Vastus lateralis skinfold thickness (13.7 ± 6.6 mm) was also assessed before placing the NIRS instrument to avoid disturbance linked to adipose tissue thickness.

#### 2.3.2. Ankle-Brachial Index (ABI)

The ABI was calculated as the ratio of the highest SBP value of posterior and dorsal tibial arteries and the highest SBP value of the brachial artery. Values are reported in Table 1. The assessment order was the following: right brachial artery; right tibial posterior and anterior arteries; left tibial posterior and anterior arteries; left brachial artery; right brachial artery [30]. SBP was assessed with a blood pressure monitor (Easy 3, Holtex+, Aix-en-Provence, France) and a manual stethoscope (Classic III, 3M Littman Stethoscopes, Maplewood, MN, USA) for the first measurement of the right brachial artery and with a mini-Doppler (Sonotrax Lite, Edan Instruments Inc., Shenzhen, China) for subsequent measures.

#### 2.3.3. Near-Infrared Spectroscopy (NIRS)

A wireless NIRS device (PortaMon, Artinis, Elst, The Netherlands), connected with Bluetooth with a sampling rate of 10 Hz, was used on the left arm. This was dual-wavelength (760 and 850 nm), with three pairs of LEDs spaced 30, 35, and 40 mm from the receiving continuous-wave NIRS system using the modified Lambert–Beer law. It calculates the absolute concentration of tissue oxy-, deoxy- and total hemoglobin (O_2_Hb, HHb, tHb, respectively). Tissue saturation index (TSI), expressed in % and reflecting the dynamic balance between O_2_ supply and consumption, was calculated as stated in Equation (1):

(1) $$\mathrm{TSI}=\left[O_{2}\mathrm{Hb}\right]/\left(\left[O_{2}\mathrm{Hb}\right]+\left[\mathrm{HHb}\right]\right)\times 100$$

**Figure 2:**
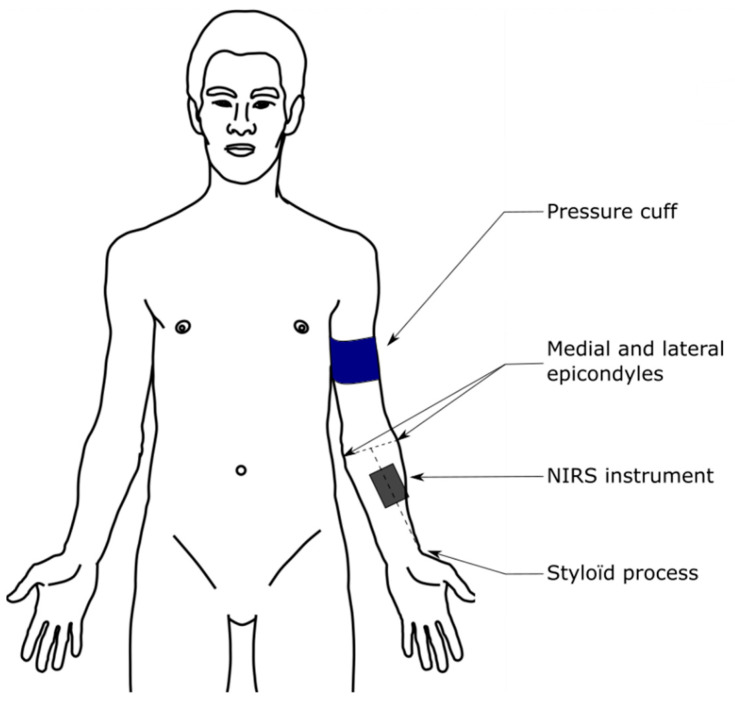
NIRS device placement illustration.

On the arm, the NIRS device was placed on the brachioradialis muscle, at two-thirds on the line from the styloid process to the central point between the lateral and medial epicondyles (Figure 2) in order to make device placement consistent for all participants [31].

The device was adhered to the limb with double-sided auto-adhesive tape (Coheban, 3M, Cergy-Pontoise, France) and wrapped in black cloth and an elastic bandage to prevent any disturbance due to light interference or unintentional movement.

### 2.4. Data Analysis

NIRS data were acquired at 10 Hz, and the signal was smoothed using a 10th-order low-pass zero-phase Butterworth filter (cut-off frequency 0.8 Hz) using Pandas software library functions for Python (Python 3.8.8, Python Software Foundation, https://www.python.org, accessed on 19 October 2021) [32]. To avoid any disturbance linked to the beginning of the protocol, TSI_baseline_, HHB_baseline_ and O_2_HB_baseline_ were calculated as the average of the last minute before the first occlusion.

**Figure 3:**
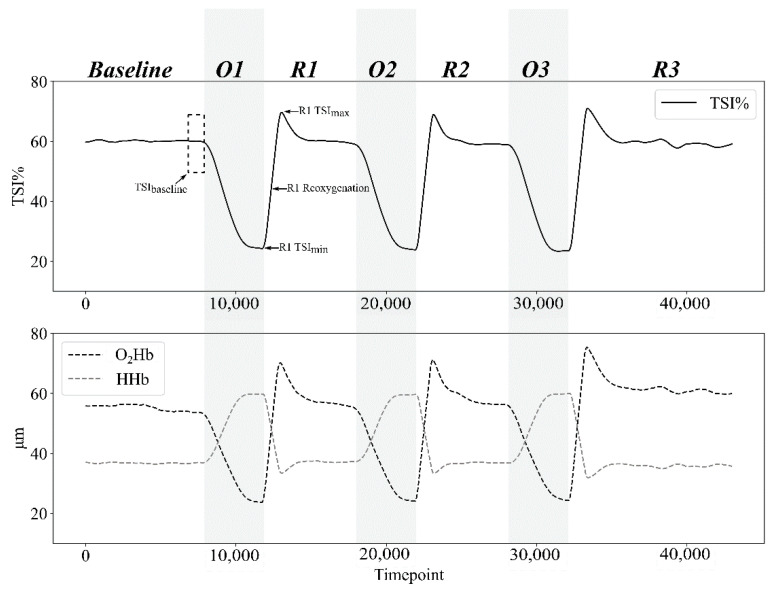
Example of tissue saturation index (TSI%), oxyhemoglobin concentration ([O_2_Hb]) and deoxyhemoglobin concentration ([HHb]) responses during the whole protocol for a single participant in group 3 (G3: 250 mmHg). The dashed rectangle represents the last minute of the baseline, which is averaged to provide the baseline value of each parameter. When the cuff is inflated during occlusion phases (O1, O2, O3), both TSI% and [O_2_Hb] decrease until their minimum, whereas [HHb] increases to its maximum. When the cuff is deflated at the beginning of reperfusions phases (R1, R2, R3), TSI% and [O_2_Hb] rise until their maximum above the baseline value (hyperemia spike), whereas [HHb] reach its minimum.

The difference (∆TSI, ∆HHB and ∆O_2_HB) between the maximum reached corresponding to the hyperemia spike during the reoxygenation (for TSI_max_ and O_2_HB_max_) and the minimum reached during the occlusion (TSI_min_ and O_2_HB_min_) and inversely for HHB_max_ and HHB_min_ were calculated (Figure 3).

Hemoglobin difference (HB_diff_) was calculated as the difference between oxygenated hemoglobin (O_2_Hb) and deoxygenated hemoglobin (HHb).

Reoxygenation rate was calculated for each parameter (TSI_reoxy_rate_, HHB_reoxy_rate_, O_2_HB_reoxy_rate_ and HB_diff_reoxy_rate_) as the upslope (r² = 0.968 ± 0.016) of TSI, [HHb], [O_2_HB] and HB_diff_ between the start and the end of the reoxygenation curve, as well as the intercept and the coefficient of determination (r²) (Figure 3). The start, the end, and the highest velocity value (Vpeak) of the reoxygenation curve were calculated using automatic peak detection Python routine, with a 5% threshold for both start and end, applied to speed values. Speed values were obtained by derivative collected data and smoothed using a 10th-order low-pass zero-phase Butterworth filter (cut-off frequency 0.8 Hz) (Python 3.8.8).

### 2.5. Statistical Analysis

The three reperfusions per session are named R1, R2 and R3 for reperfusion 1, 2 and 3, respectively. Sessions 1 and 2 are named S1 and S2, respectively.

Student’s *t*-tests were performed for the participants’ characteristics and environmental conditions using JASP (Version 0.16.1.0, www.jasp-stats.org, accessed on 24 March 2022) after checking normality (Shapiro–Wilk test) and the equality of variances (Levene’s test). The effect sizes (ES) are reported using Cohen’s d and interpreted as small (0.2 ≥ d > 0.5), medium (0.5 ≥ d > 0.8), and large (d ≥ 0.8) [33].

Intraclass correlation coefficient (ICC) and 95% confidence intervals (CI-95%) were calculated with the Pingouin package for Python using a two-way mixed-effects model with absolute agreement to compare repeated measures pairwise (Type 2, 1) [25]. Within-session ICC was calculated pairwise between R1–R2, R1–R3, R2–R3. Between-session ICC was calculated pairwise between the same event of each session: R1–R1, R2–R2, R3–R3. ICC [CI-95%] values less than 0.5 are indicative of poor reliability, values between 0.50 and 0.75 indicate moderate reliability, values between 0.75 and 0.90 indicate good reliability, and values greater than 0.90 indicate excellent reliability [25].

Standard error of measurement (SEM), an indicator of absolute reliability, was calculated as the standard deviation (SD) multiplied by the root mean square (RMS) of the difference of 1 and the ICC [34].

Coefficient of variation (CV) was used to assess the variability across multiple repeated measures. Within-participant CV was calculated as the mean of CV calculated over the three trials for each participant. Three CV between sessions were calculated for each participant for R1S1–R1S2, R2S1–R2S2 and R3S1–R3S2, respectively. A mean CV for each participant was then calculated, and the CV of each participant was averaged to obtain a mean between-session CV per group. Finally, a CV between participants was calculated for each trial and averaged.

## 3. Results

### 3.1. Participants’ Characteristics and Environmental Conditions

Non-significant differences were observed across all variables of participants’ characteristics. Comparisons of temperature and humidity level between sessions showed a significant difference between means only for humidity level (t(30) = −2.580; *p* = 0.015; ES = −0.463). Participant characteristics are reported in Table 1. Environmental conditions were monitored during each session and are reported in Table 2.

### 3.2. Within-Participant Reliability

#### 3.2.1. Maximum and Minimum Responses

Results of within-participant variability assessed with CV are reported in Table 3. Results showed a lower mean CV between trials of each session across all variables for maximums (2.58 ± 1.82%) compared with minimums (5.14 ± 3.81%). The lowest variability for maximums was reached by TSI% (1.23 ± 0.62%), whereas the lowest variability was reached by HHb (2.30 ± 0.77%) for minimums.

Reliability between measurements assessed with ICC for minimums and maximums are reported in Table 4 and Table 5, respectively. For both minimum and maximum values, ICC [ICC [CI-95%] showed a significant *p*-value for all conditions and all parameters (*p <* 0.001). Good to excellent reliability (0.75 > ICC [CI-95%] > 1) was found in all conditions for maximums and minimums of G2 and G3, for TSI% and HHb only.

#### 3.2.2. Reoxygenation Rate

Reoxygenation rate within-participant variability results are reported in Table 6. In all conditions and for all parameters, G1 showed the lowest slope value (0.50 ± 0.16%·s^−1^) compared with G2 (1.26 ± 0.17%·s^−1^) and G3 (1.62 ± 0.51%·s^−1^). The greatest Vpeak for TSI% was obtained in G3 (2.87 ± 0.67%·s^−1^), whereas G2 and G1 elicited, respectively, 2.24 ± 0.66%·s^−1^ and 1.07 ± 0.38%·s^−1^, respectively.

Despite the mean ICC value showing good reliability for TSI% (0.79 ± 0.18) and a *p*-value < 0.001 in all conditions, ICC [CI-95%] results of slope and Vpeak were not able to reach the moderate threshold of 0.5, in most conditions or parameters (Table 7 and Table 8).

#### 3.2.3. Maximums and Minimums

Results of maximum and minimum variability between sessions, assessed with CV, are reported in Table 9. When averaged across groups, CV of HHb (3.70 ± 2.95%) and TSI% (2.43 ± 2.48%) show the lowest variability for minimums and maximums, respectively. Averaged across all groups and variables, minimums and maximums showed a CV of 7.08 ± 7.17% and 4.30 ± 3.95%, respectively.

**Figure 4:**
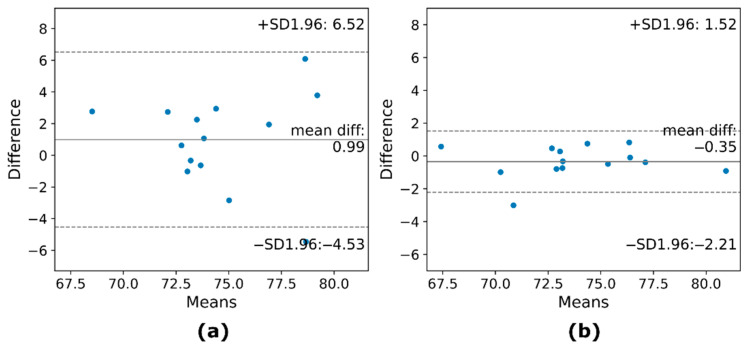
Examples of Bland–Altman plots of the reliability across trials for TSI_max_ of participants belonging to the group 2 (G2): (**a**) between sessions (bias = 0.99, 95% CI = −4.53 to 6.52) (**b**) and within sessions (bias = −0.35, 95% CI = −2.21 to 1.52). The within-session trial comparison (**b**) shows a lower dispersion around the mean compared to between-session trial comparison (**a**).

ICC [CI-95%] results for between-session analyses are reported in Table 10 and Table 11. Results indicate a lack of reliability for both maximums and minimums between sessions, with the majority of CI-95% below the 0.5 threshold in all conditions. An example of the reliability of maximums both between- and within-session is provided by an analysis of the limits of agreement (Figure 4).

#### 3.2.4. Reoxygenation Rate

Between-session variability of the reoxygenation rate parameters is reported in Table 12. For both slope and Vpeak, G1 showed CVs averaged across all variables of 41.60 ± 51.77% and 22.21 ± 32.79%, respectively, which are higher than the CVs reported for G2 (22.21 ± 32.79% and 13.07 ± 9.62%, respectively) and G3 (12.56 ± 16.40% and 8.67 ± 4.71%, respectively).

The results of the reoxygenation rate analyses of reliability are reported in Table 13 and Table 14. For TSI%, HHb and tHb, reliability could not elicit any reliable pairwise measurements of slope or Vpeak. For O_2_Hb Vpeak, only three conditions showed moderate reliability.

## 4. Discussion

The present study aims to assess the reliability of NIRS measurements of maximum, minimum and reoxygenation rate values following three occlusions. The main finding of this study is that NIRS assessment of maximums and minimums was highly reliable across trials of a single session but was not reliable on two separate occasions. Moreover, if the 95% CI of the ICC is reported, the slope of reoxygenation and Vpeak cannot be considered reliable parameters both within and between sessions.

### 4.1. Maximums and Minimums Reliability

Between sessions, our results are in agreement with the findings of Lacroix et al. (2012) for O_2_Hb and tHb maximums [35]. Their study focused on forearm oxygenation response to occlusion. However, they had a unique 5 min occlusion at 100 mmHg over the SBP (mean: 219 ± 7 mmHg), close to our G3 pressure (250 mmHg). Lacroix et al. (2012), found an ICC of 0.63 and 0.31, whereas we reported an ICC of 0.53 and 0.40 for O_2_Hb and tHb maximums, respectively (Table 11). Unfortunately, 95% CI of ICC was not reported and the O_2_Hb maximum was considered as good, while our result reported with CIs is considered as not reliable (ICC = 0.53 [0.07, 0.81]). Additionally, the authors also reported similar between-session CVs for O_2_Hb (6.68%) compared to 5.10% in the current study.

Our findings indicate lower results for minimums and for maximums of TSI in G3 (25.57 ± 9.96% and 73.48 ± 4.89%, respectively), compared to de Oliveira et al. (2021), who found a TSI of 44.00 ± 10.39% and 79.98 ± 5.11% for the minimum and maximum, respectively. Indeed, the authors assessed the StO_2_ on a similar population of young healthy adults, utilizing the same equipment (PortaMon, Artinis, Elst, Netherlands) and occlusion pressure (250 mmHg) but on a different forearm muscle (flexor carpi radialis) and with a 5 min occlusion period. These differences, particularly for TSI_min_ responses, could be attributed to the 2 min occlusion difference between studies since the duration of occlusion induces differences in microcirculatory responses [36] and significant differences in maximums and minimums of TSI% [27]. It may also result from the spatial heterogeneity of tissue responses, which makes comparison between different muscles difficult [1,37]. Moreover, differences between findings from this study for minimums recorded at brachioradialis and those at the lower limb after 5 min of occlusion at 250 mmHg (46.2 ± 7.5%) [24] could be further explained by limb-specific variation in arterial function [38].

To our knowledge, our study is the first to investigate the intra-day and day-to-day pairwise reliability of NIRS maximums and minimums after three arterial occlusions. Since the minimum and maximum values directly influence the calculation of other parameters, such as the slope of reoxygenation or the amplitude [39,40] or the maximal physiological range [23], and have an effect on the results of index calculation, such as the area under the curve (AUC), it is essential to know the reliability of those parameters. Thus, these should be ascertained both between successive measurements within a single session and also between separate occasions, such as pre- and post-intervention. Our findings highlighted the highly acceptable reliability after arterial occlusions of maximums and minimums for intra-day measurements but suggest caution when comparing values between sessions.

### 4.2. Reliability of Reoxygenation Rates

The reoxygenation rate, or reperfusion rate, has been widely investigated, and our findings are consistent with the literature [24,27,41]. In their study, Soares et al. (2019) showed that after 5 min occlusion at 250 mmHg at the arm, the NIRS reperfusion slope calculated for the forearm (flexor digitorum superficialis muscle) showed a value of 1.77 ± 0.60%·s^−1^ for TSI% [41], which is slightly higher than our findings for G3 (1.37 ± 0.33%·s^−1^). On the flexor carpi radialis muscle, a reoxygenation rate of 1.24 ± 0.41%·s^−1^ was found after 5 min occlusion at 250 mmHg [42], and, interestingly, results on leg measurements match our findings on forearm measurements, with 1.32 ± 0.38 [24] and 1.60 ± 0.80%·s^−1^ [27] for tibialis anterior.

When compared to results found in trained participants (2.83 ± 0.48%·s^−1^) [40], our results seem to be much lower and inconsistent with previous findings, despite similar population demographics and characteristics. However, in our study, walking was considered a physical activity, contrary to the work of McLay et al. (2016). Thus, it is noteworthy that our population elicits results closer to a reperfusion slope for an untrained group (1.26 ± 0.28%·s^−1^) than for a trained group [40].

The reliability of the reperfusion slope of NIRS-derived parameters has not been widely investigated in the literature. McLay et al. (2016) reported an excellent intraday ICC (0.92) with a CV of 9 ± 4% [24]. We reported ICC values of 0.91, 0.82 and 0.92 for pairwise measurements between the three occlusions in session 2. However, we reported a higher CV of 27.59 ± 13.01%. Interestingly, between-session slope measurements have been reported to be highly reliable with ICCs of 0.88 [27] and 0.94 [24] between sessions. Those findings are high when compared to our results of 0.43, 0.43 and 0.62. Moreover, we are unable to ratify a level of good reliability between sessions for our measurements, since the 95% CI reported all elicit a lower value under the threshold of 0.50. In this way, we proffer cautiousness with a comparison of reperfusion slope measurements, particularly between sessions.

The higher variability found for intra-day reoxygenation rate measurement may be explained by the extended durations of the successive occlusion and reperfusion phases, leading to a 76 min long protocol. This duration and a resting state may induce changes in skin temperature or in blood pressure levels [43], which could affect the results.

Our findings showed a dissonance with results in the literature regarding inter-day reliability. This dissonance may be due to room temperature changes between two sessions for the same participant. Even if no significant difference was found between S1 and S2, the temperature may vary between both sessions, inducing changes in both blood pressure level and muscle oxygenation kinetics [44,45]. Another explanation may result from the blood volume changes during successive occlusions and across each session [46]. Indeed, during ischemia, the muscle blood flow increases, which affects the quantity of oxy- and deoxyhemoglobin measured with the NIRS device. Thus, these blood volume changes may bring confusion in slope measurements, and some researchers suggest correcting the NIRS signal with blood volume changes [12]. However, deoxyhemoglobin is supposed to be less sensitive to blood volume changes [47], but our findings showed that HHb was not more reliable between sessions than other parameters.

This discrepancy between our findings and that of the literature is emphasized by the way in which ICCs are reported in reliability studies focusing on NIRS-derived parameters. Following recent guidelines on selecting and reporting ICCs [25], we indicated reliability according to the lower bound value of 95% CI. However, the level of reliability (“poor”, “moderate”, “good” and “excellent”) seems to differ among guidelines [48]. In the present study, 50% of within-session and no between-session measurements of reperfusion slope for TSI% are considered reliable according to the guidelines [25]. However, if ICC was reported without the 95% CI, 89% of the within-session and 22% of the between-session measurements would have been reported as reliable. This huge difference, particularly for within-session measurements, highlights the significance of reporting the 95% CI in reliability studies.

### 4.3. The Reliability When Assessing Tissue Oxygenation

NIRS technology is also used to investigate cerebral oxygenation (fNIRS). In healthy participants, fNIRS recordings of changes in cerebral oxygenation indicate good reliability (ICC = 0.83) [49]. Initial drop amplitude recorded during an orthostatic test showed good reliability for O_2_Hb (ICC = 0.83) and for TSI% (ICC = 0.99) [50]. Once again, confidence intervals are not reported in these studies. Moreover, a good to excellent test–retest reliability is found for O_2_Hb and HHb in response to postural changes [51].

The inter-day reliability of cutaneous microvascular perfusion responses assessed using integrating-probe laser Doppler flowmetry showed acceptable reliability (ICC = 0.60; CV = 13%) PORH after 5 min occlusion [52]. When assessed with laser speckle contrast analysis (LSCI), PORH indicated a good inter-day reliability (ICC = 0.76; CV = 8%) [53].

### 4.4. Limitations

The main limitation of this study may be the inability to control the room temperature, as microcirculation is sensitive to skin temperature [54]. However, no significant differences between groups or sessions have been found.

## 5. Conclusions

In conclusion, the present study demonstrated that maximum and minimum values reached after 3 periods of 7 min occlusions are highly reliable within a single session, thus it may not be necessary to perform multiple occlusions to obtain reliable values of maximums and minimums. However, NIRS-derived parameters such as the slope of reperfusion or Vpeak during reperfusion cannot show reliable reliability when ICC is reported with 95% CI. It is thus important to be cautious when comparing values between sessions, particularly when comparing pre- and post-intervention values.

## Figures and Tables

**Table 1:** Participant characteristics.

Group	G1	G2	G3	All
Occlusion pressure (mmHg)	50	164 ± 8	250	
Age (year)	22 ± 4	22 ± 3	22 ± 4	22 ± 3
Mass (kg)	77 ± 15	73 ± 13	76 ± 7	75 ± 12
Stature (cm)	177 ± 6	176 ± 7	182 ± 8	178 ± 7
Body fat (%)	14.5 ± 5.4	16.6 ± 5.5	12.6 ± 2.7	14.6 ± 4.8
SBP (mmHg)	115 ± 7	116 ± 10	121 ± 9	118 ± 9
DBP (mmHg)	66 ± 7	71 ± 6	68 ± 7	69 ± 7
ABI	1.04 ± 0.08	1.01 ± 0.12	1.03 ± 0.06	1.03 ± 0.09

Legend: Values are reported as mean ± SD; SBP: Systolic Blood Pressure; DBP: Diastolic Blood Pressure; ABI: Ankle to Brachial Index.

**Table 2:** Environmental conditions between sessions.

Group	G1	G2	G3	All
Temperature S1 (°C)	21 ± 1	22 ± 2	21 ± 2	21 ± 2
Temperature S2 (°C)	21 ± 1	22 ± 2	21 ± 1	21 ± 1
Humidity level S1 (%)	35 ± 13	43 ± 14	40 ± 13	40 ± 13 ⸸
Humidity level S2 (%)	40 ± 15	48 ± 14	45 ± 15	45 ± 14 ⸸

Legend: Values are reported as mean ± SD; S1: session 1; S2: session 2; ⸸: significative difference between S1 and S2 (*p* < 0.05).

**Table 3:** Maximum and minimum variability.

Variable	Group	Mean	SD	Mean CV Btw. Trials (%)	Mean SD Btw. Trials (%)	CV Mean (%)	CV SD (%)
TSI_min_	G1	53.61	4.74	2.14	0.18	8.83	1.76
(%)	G2	31.41	9.76	4.89	0.90	31.13	4.48
	G3	25.57	9.96	6.01	1.01	39.05	4.01
O_2_Hb_min_	G1	59.74	8.65	3.25	0.51	14.48	0.64
(µM)	G2	38.97	13.73	6.08	0.74	35.20	1.68
	G3	39.12	12.31	5.77	0.04	31.49	3.26
HHb_min_	G1	31.41	4.13	3.39	0.14	13.14	2.14
(µM)	G2	32.59	5.30	1.78	0.14	16.27	1.14
	G3	33.60	6.51	1.32	0.22	19.39	2.96
tHb_min_	G1	92.14	11.98	2.04	0.38	13.02	1.57
(µM)	G2	99.10	16.75	2.67	0.40	16.87	1.96
	G3	101.06	17.05	3.01	0.20	16.88	0.99
TSI_max_	G1	64.70	4.60	2.02	0.07	7.12	0.78
(%)	G2	75.35	4.88	0.95	0.09	6.47	0.47
	G3	73.48	4.89	0.72	0.07	6.68	2.26
O_2_Hb_max_	G1	75.83	16.58	2.86	0.22	21.86	0.66
(µM)	G2	81.25	10.96	2.13	0.00	13.49	1.30
	G3	82.73	12.37	1.98	0.16	14.97	1.39
HHb_max_	G1	50.90	8.02	3.11	0.91	15.74	2.92
(µM)	G2	63.14	9.88	1.85	0.26	15.62	1.76
	G3	64.94	9.52	1.94	0.31	14.65	0.35
tHb_max_	G1	126.61	23.18	2.01	0.66	18.31	0.34
(µM)	G2	117.94	16.79	2.07	0.38	14.23	1.43
	G3	120.88	17.35	1.67	0.27	14.36	0.23

Legend: TSI: tissue saturation index; O_2_Hb: oxyhemoglobin; HHb: deoxyhemoglobin, tHb: total hemoglobin; CV: coefficient of variation; SD: standard deviation; btw: between. CV mean and CV SD between trials are calculated for the same participant and averaged per group.

**Table 4:** Within-participant reliability for minimums.

Variable	Condition	SEM	ICC [CI-95%]	F
TSI	G1_S1_R1_R2	2.00	0.88 [0.62, 0.97] *	16.22
(%)	G1_S1_R1_R3	1.56	0.92 [0.72, 0.98] *	22.43
	G1_S1_R2_R3	1.33	0.92 [0.66, 0.98] *	34.25
	G1_S2_R1_R2	0.61	0.97 [0.86, 0.99] **	100.52
	G1_S2_R1_R3	1.58	0.84 [0.5, 0.96] *	11.04
	G1_S2_R2_R3	1.49	0.87 [0.54, 0.96] *	12.57
	G2_S1_R1_R2	0.97	0.99 [0.97, 1.0] ***	228.17
	G2_S1_R1_R3	2.36	0.95 [0.86, 0.98] **	36.81
	G2_S1_R2_R3	1.99	0.96 [0.9, 0.99] **	51.42
	G2_S2_R1_R2	1.47	0.97 [0.92, 0.99] ***	71.26
	G2_S2_R1_R3	2.06	0.95 [0.85, 0.98] **	34.30
	G2_S2_R2_R3	1.27	0.98 [0.94, 0.99] ***	88.07
	G3_S1_R1_R2	1.16	0.99 [0.96, 1.0] ***	145.88
	G3_S1_R1_R3	1.43	0.98 [0.94, 0.99] ***	92.65
	G3_S1_R2_R3	1.36	0.98 [0.95, 0.99] ***	103.27
	G3_S2_R1_R2	1.74	0.97 [0.91, 0.99] ***	59.02
	G3_S2_R1_R3	1.88	0.96 [0.89, 0.99] **	47.02
	G3_S2_R2_R3	1.26	0.98 [0.94, 0.99] ***	101.32
O_2_Hb	G1_S1_R1_R2	1.83	0.95 [0.79, 0.99] **	49.63
(µM)	G1_S1_R1_R3	2.96	0.87 [0.58, 0.97] *	14.09
	G1_S1_R2_R3	1.51	0.97 [0.88, 0.99] **	58.16
	G1_S2_R1_R2	2.40	0.92 [0.72, 0.98] *	22.69
	G1_S2_R1_R3	3.88	0.81 [0.43, 0.95]	10.95
	G1_S2_R2_R3	1.91	0.95 [0.61, 0.99] *	65.55
	G2_S1_R1_R2	4.57	0.9 [0.74, 0.97] *	19.18
	G2_S1_R1_R3	3.81	0.93 [0.81, 0.98] **	26.74
	G2_S1_R2_R3	2.41	0.97 [0.92, 0.99] ***	65.26
	G2_S2_R1_R2	3.39	0.93 [0.73, 0.98] *	40.33
	G2_S2_R1_R3	2.50	0.96 [0.82, 0.99] **	65.90
	G2_S2_R2_R3	2.10	0.97 [0.92, 0.99] ***	69.04
	G3_S1_R1_R2	3.57	0.92 [0.78, 0.97] **	23.18
	G3_S1_R1_R3	2.08	0.98 [0.93, 0.99] ***	79.64
	G3_S1_R2_R3	3.03	0.94 [0.82, 0.98] **	28.75
	G3_S2_R1_R2	2.08	0.97 [0.91, 0.99] ***	56.58
	G3_S2_R1_R3	3.01	0.93 [0.8, 0.97] **	26.94
	G3_S2_R2_R3	3.07	0.93 [0.81, 0.98] **	27.33
HHb	G1_S1_R1_R2	0.81	0.96 [0.84, 0.99] **	43.09
(µM)	G1_S1_R1_R3	1.24	0.87 [0.55, 0.97] *	16.55
	G1_S1_R2_R3	1.52	0.82 [0.45, 0.95]	10.07
	G1_S2_R1_R2	0.58	0.98 [0.94, 1.0] ***	110.64
	G1_S2_R1_R3	1.65	0.86 [0.54, 0.96] *	15.14
	G1_S2_R2_R3	1.42	0.9 [0.62, 0.97] *	22.49
	G2_S1_R1_R2	0.92	0.97 [0.92, 0.99] ***	73.30
	G2_S1_R1_R3	0.96	0.97 [0.92, 0.99] ***	66.34
	G2_S1_R2_R3	0.42	0.99 [0.98, 1.0] ***	309.94
	G2_S2_R1_R2	0.84	0.97 [0.91, 0.99] ***	83.32
	G2_S2_R1_R3	0.94	0.96 [0.84, 0.99] **	78.49
	G2_S2_R2_R3	0.50	0.99 [0.97, 1.0] ***	210.56
	G3_S1_R1_R2	0.33	1 [0.99, 1.0] ***	1321.88
	G3_S1_R1_R3	0.53	0.99 [0.98, 1.0] ***	339.18
	G3_S1_R2_R3	0.58	0.99 [0.98, 1.0] ***	275.76
	G3_S2_R1_R2	0.61	0.99 [0.97, 1.0] ***	171.15
	G3_S2_R1_R3	0.71	0.98 [0.95, 0.99] ***	143.09
	G3_S2_R2_R3	0.45	0.99 [0.98, 1.0] ***	349.01
tHb	G1_S1_R1_R2	2.16	0.96 [0.86, 0.99] **	57.42
(µM)	G1_S1_R1_R3	2.28	0.95 [0.83, 0.99] **	38.64
	G1_S1_R2_R3	1.37	0.98 [0.94, 1.0] ***	144.38
	G1_S2_R1_R2	2.44	0.96 [0.86, 0.99] **	48.62
	G1_S2_R1_R3	3.52	0.92 [0.74, 0.98] *	24.80
	G1_S2_R2_R3	1.64	0.98 [0.93, 1.0] ***	120.85
	G2_S1_R1_R2	2.85	0.98 [0.93, 0.99] ***	77.17
	G2_S1_R1_R3	3.28	0.97 [0.91, 0.99] ***	60.21
	G2_S1_R2_R3	2.13	0.99 [0.96, 1.0] ***	141.39
	G2_S2_R1_R2	4.83	0.89 [0.7, 0.96] *	19.56
	G2_S2_R1_R3	3.51	0.94 [0.68, 0.98] *	55.99
	G2_S2_R2_R3	3.32	0.95 [0.86, 0.98] **	36.74
	G3_S1_R1_R2	3.85	0.95 [0.86, 0.98] **	36.88
	G3_S1_R1_R3	2.41	0.98 [0.95, 0.99] ***	104.67
	G3_S1_R2_R3	3.95	0.94 [0.84, 0.98] **	31.51
	G3_S2_R1_R2	3.63	0.95 [0.87, 0.98] **	40.01
	G3_S2_R1_R3	3.83	0.94 [0.84, 0.98] **	33.40
	G3_S2_R2_R3	3.29	0.96 [0.88, 0.99] **	44.93

Legend: G1: group 1; G2: group 2; G3: group 3; S1: session 1; S2: session 2; R1: first reperfusion; R2: second reperfusion; R3: third reperfusion. Standard error of measurement (SEM) is expressed in arbitrary units (u.a.). Intraclass correlation coefficient (ICC) type: (2, 1); Degrees of freedom (df): 9, 14, 14 for G1, G2 and G3, respectively. *p* < 0.001 in all conditions. * Moderate reliability; ** Good reliability; *** Excellent reliability.

**Table 5:** Within-participant reliability for maximums.

Variable	Condition	SEM	ICC [CI-95%]	F
TSI	G1_S1_R1_R2	0.83	0.97 [0.89, 0.99] **	88.24
(%)	G1_S1_R1_R3	1.82	0.85 [0.54, 0.96] *	14.10
	G1_S1_R2_R3	1.59	0.88 [0.6, 0.97] *	14.84
	G1_S2_R1_R2	0.59	0.98 [0.92, 0.99] ***	85.33
	G1_S2_R1_R3	1.70	0.84 [0.52, 0.96] *	12.73
	G1_S2_R2_R3	1.76	0.84 [0.49, 0.96]	13.01
	G2_S1_R1_R2	1.30	0.92 [0.79, 0.97] **	24.12
	G2_S1_R1_R3	0.97	0.96 [0.89, 0.99] **	47.76
	G2_S1_R2_R3	0.81	0.96 [0.88, 0.99] **	61.66
	G2_S2_R1_R2	1.05	0.96 [0.88, 0.99] **	44.38
	G2_S2_R1_R3	1.42	0.92 [0.8, 0.97] **	26.09
	G2_S2_R2_R3	0.60	0.98 [0.95, 0.99] ***	139.95
	G3_S1_R1_R2	0.65	0.99 [0.97, 1.0] ***	156.65
	G3_S1_R1_R3	0.72	0.98 [0.96, 0.99] ***	135.37
	G3_S1_R2_R3	0.40	1 [0.99, 1.0] ***	475.50
	G3_S2_R1_R2	0.76	0.96 [0.88, 0.99] **	47.45
	G3_S2_R1_R3	0.81	0.95 [0.87, 0.98] **	40.06
	G3_S2_R2_R3	0.39	0.99 [0.97, 1.0] ***	164.66
O_2_Hb	G1_S1_R1_R2	2.79	0.97 [0.89, 0.99] **	65.72
(µM)	G1_S1_R1_R3	3.48	0.96 [0.84, 0.99] **	42.47
	G1_S1_R2_R3	1.48	0.99 [0.97, 1.0] ***	242.21
	G1_S2_R1_R2	1.23	0.99 [0.97, 1.0] ***	285.16
	G1_S2_R1_R3	3.04	0.96 [0.81, 0.99] **	71.85
	G1_S2_R2_R3	3.09	0.96 [0.76, 0.99] **	70.93
	G2_S1_R1_R2	1.88	0.98 [0.91, 0.99] ***	105.41
	G2_S1_R1_R3	2.43	0.96 [0.78, 0.99] **	69.59
	G2_S1_R2_R3	0.95	0.99 [0.97, 1.0] ***	370.10
	G2_S2_R1_R2	1.50	0.98 [0.82, 0.99] **	154.37
	G2_S2_R1_R3	2.50	0.94 [0.27, 0.99]	90.95
	G2_S2_R2_R3	1.51	0.98 [0.84, 0.99] **	143.04
	G3_S1_R1_R2	1.51	0.99 [0.94, 1.0] ***	178.14
	G3_S1_R1_R3	2.22	0.97 [0.72, 0.99] *	133.93
	G3_S1_R2_R3	1.64	0.98 [0.94, 0.99] ***	150.76
	G3_S2_R1_R2	2.27	0.96 [0.89, 0.99] **	50.78
	G3_S2_R1_R3	2.34	0.96 [0.88, 0.99] **	53.42
	G3_S2_R2_R3	1.37	0.99 [0.96, 1.0] ***	144.53
HHb	G1_S1_R1_R2	2.09	0.9 [0.26, 0.98]	42.70
(µM)	G1_S1_R1_R3	2.50	0.87 [0.5, 0.97] *	19.84
	G1_S1_R2_R3	1.52	0.95 [0.81, 0.99] **	34.38
	G1_S2_R1_R2	1.20	0.98 [0.93, 1.0] ***	122.78
	G1_S2_R1_R3	1.70	0.96 [0.71, 0.99] *	92.60
	G1_S2_R2_R3	1.33	0.98 [0.91, 0.99] ***	104.52
	G2_S1_R1_R2	1.59	0.98 [0.93, 0.99] ***	82.03
	G2_S1_R1_R3	1.52	0.98 [0.94, 0.99] ***	94.54
	G2_S1_R2_R3	1.06	0.99 [0.97, 1.0] ***	187.16
	G2_S2_R1_R2	1.35	0.98 [0.82, 0.99] **	148.52
	G2_S2_R1_R3	1.39	0.97 [0.91, 0.99] ***	100.13
	G2_S2_R2_R3	0.58	1 [0.99, 1.0] ***	494.12
	G3_S1_R1_R2	1.44	0.98 [0.93, 0.99] ***	77.85
	G3_S1_R1_R3	1.11	0.99 [0.96, 0.99] ***	124.83
	G3_S1_R2_R3	1.25	0.98 [0.94, 0.99] ***	94.77
	G3_S2_R1_R2	1.42	0.98 [0.94, 0.99] ***	91.88
	G3_S2_R1_R3	1.61	0.97 [0.92, 0.99] ***	68.96
	G3_S2_R2_R3	1.56	0.97 [0.92, 0.99] ***	67.93
tHb	G1_S1_R1_R2	3.22	0.98 [0.91, 0.99] ***	112.34
(µM)	G1_S1_R1_R3	4.78	0.96 [0.84, 0.99] **	48.67
	G1_S1_R2_R3	3.02	0.98 [0.93, 1.0] ***	103.70
	G1_S2_R1_R2	1.90	0.99[0.97, 1.0] ***	319.43
	G1_S2_R1_R3	2.43	0.99 [0.96, 1.0] ***	165.32
	G1_S2_R2_R3	3.51	0.98 [0.91, 0.99] ***	87.44
	G2_S1_R1_R2	4.47	0.94 [0.83, 0.98] **	30.94
	G2_S1_R1_R3	3.74	0.96 [0.88, 0.99] **	47.94
	G2_S1_R2_R3	1.89	0.99 [0.96, 1.0] ***	191.37
	G2_S2_R1_R2	2.11	0.98 [0.68, 1.0] *	252.60
	G2_S2_R1_R3	2.88	0.96 [0.43, 0.99]	153.16
	G2_S2_R2_R3	1.44	0.99 [0.97, 1.0] ***	271.99
	G3_S1_R1_R2	2.01	0.99 [0.95, 1.0] ***	167.18
	G3_S1_R1_R3	2.47	0.98 [0.77, 0.99] **	192.52
	G3_S1_R2_R3	2.05	0.98 [0.95, 0.99] ***	139.59
	G3_S2_R1_R2	2.99	0.97 [0.92, 0.99] ***	76.27
	G3_S2_R1_R3	2.62	0.98 [0.93, 0.99] ***	102.32
	G3_S2_R2_R3	2.82	0.97 [0.92, 0.99] ***	71.31

Legend: G1: group 1; G2: group 2; G3: group 3; S1: session 1; S2: session 2; R1: first reperfusion; R2: second reperfusion; R3: third reperfusion. Standard error of measurement (SEM) is expressed in arbitrary units (u.a.). Intraclass correlation coefficient (ICC) type: (2, 1); df: 9, 14, 14 for G1, G2 and G3, respectively. *p* < 0.001 in all conditions. * Moderate reliability; ** Good reliability; *** Excellent reliability.

**Table 6:** Reoxygenation rate within-participant variability.

Variable	Groupe	Mean	SD	Mean CV Btw. Trials (%)	Mean SD Btw. Trials (%)	Mean CV (%)	Mean SD (%)
TSI_Slope_	G1	0.53	0.15	107.50	130.42	52.56	19.85
(%·s^−1^)	G2	1.29	0.02	9.21	1.21	36.69	1.62
	G3	1.66	0.05	11.10	1.15	27.59	13.01
O_2_Hb_Slope_	G1	0.44	0.26	65.47	59.61	107.39	58.25
(µM·s^−1^)	G2	1.37	0.08	16.11	3.72	37.44	2.55
	G3	1.75	0.08	12.49	1.50	27.29	3.58
HHb_Slope_	G1	0.52	0.13	17.51	9.62	58.26	0.76
(µM·s^−1^)	G2	1.07	0.14	12.17	2.63	32.73	3.85
	G3	1.31	0.06	13.48	1.14	25.56	3.91
tHb_Slope_	G1	0.51	0.24	33.25	19.46	82.36	31.64
(µM·s^−1^)	G2	1.29	0.27	33.55	5.18	62.29	26.41
	G3	1.76	0.10	24.72	3.59	47.04	10.47
TSI_Vpeak_	G1	1.07	0.38	13.89	5.37	35.78	0.62
(%·s^−1^)	G2	2.24	0.66	9.37	0.52	29.35	2.58
	G3	2.87	0.67	9.57	0.37	23.33	11.13
O_2_Hb_Vpeak_	G1	2.82	2.05	9.47	0.82	73.36	7.75
(µM·s^−1^)	G2	2.58	0.89	13.05	0.46	34.68	0.07
	G3	2.90	0.87	15.30	2.11	30.18	0.62
HHb_Vpeak_	G1	2.11	0.95	7.09	0.46	45.16	2.13
(µM·s^−1^)	G2	1.74	0.57	10.76	0.02	33.08	2.20
	G3	2.02	0.59	11.06	0.36	29.61	2.95
tHb_Vpeak_	G1	4.85	2.97	6.92	2.03	61.38	4.21
(µM·s^−1^)	G2	2.37	0.81	19.42	2.20	34.15	2.45
	G3	2.39	0.72	21.27	2.10	30.38	8.36

Legend: TSI: tissue saturation index; O_2_Hb: oxyhemoglobin; HHb: deoxyhemoglobin, tHb: total hemoglobin. CV: coefficient of variation; SD: standard deviation; btw: between. CV mean and CV SD between trials are calculated for the same participant and averaged per group.

**Table 7:** Within-participant reliability for slope of reoxygenation.

Variable	Condition	SEM	ICC [CI-95%]	F
TSI	G1_S1_R1_R2	0.25	0.38 [−0.20, 0.80]	2.48
(%·s^−1^)	G1_S1_R1_R3	0.25	0.37 [−0.22, 0.80]	2.37
	G1_S1_R2_R3	0.09	0.75 [0.21, 0.94]	6.49
	G1_S2_R1_R2	0.07	0.91 [0.63, 0.98] *	28.22
	G1_S2_R1_R3	0.13	0.67 [0.02, 0.92]	4.60
	G1_S2_R2_R3	0.10	0.74 [0.25, 0.93]	6.91
	G2_S1_R1_R2	0.09	0.96 [0.87, 0.99] **	55.42
	G2_S1_R1_R3	0.15	0.89 [0.54, 0.97] *	27.53
	G2_S1_R2_R3	0.11	0.94 [0.81, 0.98] **	42.94
	G2_S2_R1_R2	0.12	0.94 [0.46, 0.98]	63.09
	G2_S2_R1_R3	0.14	0.92 [0.19, 0.98]	66.09
	G2_S2_R2_R3	0.12	0.94 [0.83, 0.98] **	32.28
	G3_S1_R1_R2	0.17	0.73 [−0.08, 0.94]	24.98
	G3_S1_R1_R3	0.19	0.63 [−0.09, 0.90]	14.93
	G3_S1_R2_R3	0.11	0.82 [0.53, 0.94] *	10.05
	G3_S2_R1_R2	0.18	0.91 [0.74, 0.97] *	21.78
	G3_S2_R1_R3	0.23	0.82 [0.50, 0.94] *	11.85
	G3_S2_R2_R3	0.17	0.92 [0.76, 0.97] **	22.97
O_2_Hb	G1_S1_R1_R2	0.08	0.95 [0.80, 0.99] **	39.40
(µM·s^−1^)	G1_S1_R1_R3	0.18	0.72 [0.16, 0.93]	7.82
	G1_S1_R2_R3	0.13	0.83 [0.43, 0.96]	13.19
	G1_S2_R1_R2	0.10	0.93 [0.72, 0.98] *	25.10
	G1_S2_R1_R3	0.14	0.86 [0.53, 0.97] *	15.09
	G1_S2_R2_R3	0.12	0.91 [0.63, 0.98] *	25.71
	G2_S1_R1_R2	0.34	0.67 [0.22, 0.88]	6.29
	G2_S1_R1_R3	0.42	0.56 [0.03, 0.84]	4.89
	G2_S1_R2_R3	0.15	0.91 [0.67, 0.97] *	28.21
	G2_S2_R1_R2	0.16	0.88 [0.49, 0.96]	23.29
	G2_S2_R1_R3	0.24	0.76 [0.05, 0.93]	14.68
	G2_S2_R2_R3	0.18	0.88 [0.63, 0.96] *	18.25
	G3_S1_R1_R2	0.02	0.8 [−0.03, 0.95]	24.74
	G3_S1_R1_R3	0.02	0.69 [−0.08, 0.92]	15.13
	G3_S1_R2_R3	0.01	0.92 [0.77, 0.98] **	26.16
	G3_S2_R1_R2	0.03	0.69 [0.14, 0.90]	7.95
	G3_S2_R1_R3	0.02	0.78 [0.13, 0.94]	14.44
	G3_S2_R2_R3	0.02	0.88 [0.66, 0.96] *	15.05
HHb	G1_S1_R1_R2	0.06	0.93 [0.74, 0.98] *	31.39
(µM·s^−1^)	G1_S1_R1_R3	0.13	0.67 [0.05, 0.91]	7.17
	G1_S1_R2_R3	0.09	0.81 [0.28, 0.95]	13.33
	G1_S2_R1_R2	0.05	0.98 [0.92, 1.00] ***	97.22
	G1_S2_R1_R3	0.06	0.97 [0.84, 0.99] **	71.73
	G1_S2_R2_R3	0.06	0.97 [0.88, 0.99] **	63.89
	G2_S1_R1_R2	0.11	0.9 [0.71, 0.97] *	20.99
	G2_S1_R1_R3	0.15	0.82 [0.53, 0.94] *	10.84
	G2_S1_R2_R3	0.12	0.88 [0.68, 0.96] *	15.34
	G2_S2_R1_R2	0.13	0.84 [0.56, 0.95] *	13.40
	G2_S2_R1_R3	0.16	0.76 [0.19, 0.93]	11.96
	G2_S2_R2_R3	0.13	0.86 [0.62, 0.95] *	14.29
	G3_S1_R1_R2	0.19	0.74 [0.34, 0.91]	7.84
	G3_S1_R1_R3	0.25	0.55 [0.05, 0.84]	4.35
	G3_S1_R2_R3	0.19	0.72 [0.32, 0.91]	6.15
	G3_S2_R1_R2	0.14	0.75 [0.37, 0.92]	8.15
	G3_S2_R1_R3	0.19	0.71 [0.12, 0.91]	9.08
	G3_S2_R2_R3	0.17	0.74 [0.36, 0.91]	7.01
tHb	G1_S1_R1_R2	0.09	0.93 [0.73, 0.98] *	26.50
(µM·s^−1^)	G1_S1_R1_R3	0.16	0.7 [0.17, 0.92]	6.62
	G1_S1_R2_R3	0.13	0.8 [0.37, 0.95]	9.14
	G1_S2_R1_R2	0.05	0.98 [0.93, 1.0] ***	103.74
	G1_S2_R1_R3	0.12	0.89 [0.58, 0.97] *	20.10
	G1_S2_R2_R3	0.14	0.87 [0.54, 0.97] *	14.17
	G2_S1_R1_R2	0.26	0.91 [0.75, 0.97] *	22.62
	G2_S1_R1_R3	0.26	0.92 [0.77, 0.98] **	29.02
	G2_S1_R2_R3	0.22	0.93 [0.81, 0.98] **	28.05
	G2_S2_R1_R2	0.34	0.74 [0.30, 0.91]	9.04
	G2_S2_R1_R3	0.30	0.83 [0.45, 0.95]	15.42
	G2_S2_R2_R3	0.24	0.86 [0.63, 0.95] *	12.87
	G3_S1_R1_R2	0.19	0.91 [0.24, 0.98]	53.41
	G3_S1_R1_R3	0.53	0.11 [−0.25, 0.54]	1.35
	G3_S1_R2_R3	0.52	0.1 [−0.41, 0.59]	1.24
	G3_S2_R1_R2	0.48	0.76 [0.41, 0.92]	7.94
	G3_S2_R1_R3	0.41	0.81 [0.50, 0.94] *	10.23
	G3_S2_R2_R3	0.24	0.93 [0.79, 0.98] **	25.95

Legend: G1: group 1; G2: group 2; G3: group 3; S1: session 1; S2: session 2; R1: first reperfusion; R2: second reperfusion; R3: third reperfusion. Standard error of measurement (SEM) is expressed in arbitrary units (u.a.). Intraclass correlation coefficient (ICC) type: (2, 1); df: 8, 13, 12 for G1, G2 and G3, respectively. *p* < 0.001 in all conditions. * Moderate reliability; ** Good reliability; *** Excellent reliability.

**Table 8:** Within-participant reliability for Vpeak of reoxygenation rate.

Variable	Condition	SEM	ICC [CI-95%]	F
TSI%	G1_S1_R1_R2	0.10	0.92 [0.69, 0.98] *	21.72
(%·s^−1^)	G1_S1_R1_R3	0.20	0.51 [−0.24, 0.87]	2.84
	G1_S1_R2_R3	0.18	0.66 [0.01, 0.91]	4.48
	G1_S2_R1_R2	0.07	0.97 [0.88, 0.99] **	68.24
	G1_S2_R1_R3	0.16	0.82 [0.38, 0.96]	9.12
	G1_S2_R2_R3	0.11	0.9 [0.61, 0.98] *	16.53
	G2_S1_R1_R2	0.20	0.89 [0.23, 0.97]	40.40
	G2_S1_R1_R3	0.30	0.8 [0.12, 0.94]	17.29
	G2_S1_R2_R3	0.16	0.94 [0.81, 0.98] **	33.87
	G2_S2_R1_R2	0.18	0.93 [0.61, 0.98] *	43.94
	G2_S2_R1_R3	0.23	0.89 [0.30, 0.97]	33.26
	G2_S2_R2_R3	0.17	0.94 [0.82, 0.98] **	30.46
	G3_S1_R1_R2	0.31	0.58 [−0.09, 0.89]	14.37
	G3_S1_R1_R3	0.30	0.59 [−0.09, 0.89]	14.57
	G3_S1_R2_R3	0.09	0.96 [0.87, 0.99] **	45.44
	G3_S2_R1_R2	0.36	0.84 [0.54, 0.95] *	13.70
	G3_S2_R1_R3	0.24	0.9 [0.69, 0.97] *	23.71
	G3_S2_R2_R3	0.29	0.87 [0.63, 0.96] *	13.78
O_2_Hb	G1_S1_R1_R2	0.46	0.95 [0.82, 0.99] **	41.27
(µM·s^−1^)	G1_S1_R1_R3	0.37	0.97 [0.88, 0.99] **	65.49
	G1_S1_R2_R3	0.15	0.99 [0.98, 1.0] ***	322.77
	G1_S2_R1_R2	0.21	0.99 [0.95, 1.0] ***	147.02
	G1_S2_R1_R3	0.16	0.99 [0.97, 1.0] ***	303.78
	G1_S2_R2_R3	0.23	0.99 [0.94, 1.0] ***	152.72
	G2_S1_R1_R2	0.34	0.87 [0.03, 0.97]	42.00
	G2_S1_R1_R3	0.51	0.75 [0.02, 0.93]	14.39
	G2_S1_R2_R3	0.26	0.93 [0.79, 0.98] **	33.23
	G2_S2_R1_R2	0.34	0.85 [0.55, 0.95] *	14.94
	G2_S2_R1_R3	0.38	0.84 [0.04, 0.96]	29.73
	G2_S2_R2_R3	0.33	0.86 [0.62, 0.95] *	15.05
	G3_S1_R1_R2	0.45	0.74 [0.11, 0.93]	11.55
	G3_S1_R1_R3	0.57	0.7 [−0.07, 0.92]	14.50
	G3_S1_R2_R3	0.47	0.74 [0.37, 0.91]	7.22
	G3_S2_R1_R2	0.42	0.79 [−0.06, 0.96]	44.60
	G3_S2_R1_R3	0.49	0.66 [−0.09, 0.91]	13.41
	G3_S2_R2_R3	0.26	0.91 [0.72, 0.97] *	19.06
HHb	G1_S1_R1_R2	0.22	0.94 [0.79, 0.99] **	38.02
(µM·s^−1^)	G1_S1_R1_R3	0.20	0.96 [0.83, 0.99] **	45.48
	G1_S1_R2_R3	0.10	0.99 [0.95, 1.00] ***	159.37
	G1_S2_R1_R2	0.12	0.98 [0.91, 1.00] ***	148.02
	G1_S2_R1_R3	0.18	0.97 [0.87, 0.99] **	58.41
	G1_S2_R2_R3	0.16	0.97 [0.88, 0.99] **	61.26
	G2_S1_R1_R2	0.19	0.89 [0.66, 0.97] *	22.05
	G2_S1_R1_R3	0.25	0.84 [0.32, 0.96]	19.66
	G2_S1_R2_R3	0.11	0.96 [0.80, 0.99] **	76.43
	G2_S2_R1_R2	0.21	0.85 [0.17, 0.96]	26.33
	G2_S2_R1_R3	0.21	0.85 [−0.04, 0.97]	51.73
	G2_S2_R2_R3	0.16	0.92 [0.78, 0.97] **	23.74
	G3_S1_R1_R2	0.24	0.86 [0.28, 0.96]	23.63
	G3_S1_R1_R3	0.25	0.86 [0.03, 0.97]	37.29
	G3_S1_R2_R3	0.10	0.97 [0.91, 0.99] ***	67.75
	G3_S2_R1_R2	0.34	0.66 [0.07, 0.89]	7.67
	G3_S2_R1_R3	0.26	0.74 [0.04, 0.93]	12.90
	G3_S2_R2_R3	0.18	0.87 [0.64, 0.96] *	14.27
tHb	G1_S1_R1_R2	0.66	0.95 [0.83, 0.99] **	42.79
(µM·s^−1^)	G1_S1_R1_R3	0.53	0.97 [0.89, 0.99] **	70.64
	G1_S1_R2_R3	0.23	0.99 [0.97, 1.00] ***	288.36
	G1_S2_R1_R2	0.28	0.99 [0.96, 1.00] ***	202.64
	G1_S2_R1_R3	0.17	1 [0.99, 1.00] ***	534.65
	G1_S2_R2_R3	0.29	0.99 [0.95, 1.00] ***	189.00
	G2_S1_R1_R2	0.35	0.83 [0.42, 0.95]	15.31
	G2_S1_R1_R3	0.65	0.56 [0.10, 0.83]	3.82
	G2_S1_R2_R3	0.45	0.77 [0.43, 0.92]	7.38
	G2_S2_R1_R2	0.58	0.44 [−0.13, 0.78]	2.46
	G2_S2_R1_R3	0.36	0.84 [0.59, 0.95] *	11.65
	G2_S2_R2_R3	0.54	0.58 [0.08, 0.84]	3.59
	G3_S1_R1_R2	0.49	0.62 [0.17, 0.87]	4.49
	G3_S1_R1_R3	0.53	0.6 [−0.10, 0.89]	10.31
	G3_S1_R2_R3	0.67	0.4 [−0.09, 0.76]	2.56
	G3_S2_R1_R2	0.46	0.49 [−0.11, 0.83]	6.04
	G3_S2_R1_R3	0.59	0.27 [−0.14, 0.66]	2.26
	G3_S2_R2_R3	0.49	0.46 [−0.13, 0.80]	2.56

Legend: G1: group 1; G2: group 2; G3: group 3; S1: session 1; S2: session 2; R1: first reperfusion; R2: second reperfusion; R3: third reperfusion. Standard error of measurement (SEM) is expressed in arbitrary units (u.a.). Intraclass correlation coefficient (ICC) type: (2, 1); df: 8, 13, 12 for G1, G2 and G3, respectively. *p* < 0.01 in all conditions. * Moderate reliability; ** Good reliability; *** Excellent reliability.

**Table 9:** Between-session variability for minimums and maximums.

Variable	Group	Mean_S1	SD_S1	Mean_S2	SD_S2	Mean CV Btw. Sessions (%)	SD of CV Btw. Sessions (%)
TSI_min_	G1	54.55	5.39	52.67	3.85	3.33	1.99
(%)	G2	30.79	10.45	32.02	8.87	9.11	8.34
	G3	24.75	10.30	26.38	9.49	15.05	12.17
O_2_Hb_min_	G1	60.75	8.33	58.72	8.49	4.11	2.82
(µM)	G2	40.54	14.42	37.41	12.58	9.91	5.45
	G3	38.54	12.84	39.70	11.36	13.03	9.55
HHb_min_	G1	31.78	3.61	31.04	4.44	3.60	2.77
(µM)	G2	32.44	5.50	32.75	5.03	3.14	2.11
	G3	33.26	7.13	33.94	5.85	4.33	3.75
tHb_min_	G1	93.55	11.03	90.74	12.63	3.48	2.55
(µM)	G2	101.70	18.43	96.49	14.63	5.28	3.15
	G3	99.30	17.26	102.82	16.37	6.03	4.24
TSI_max_	G1	64.02	4.78	65.39	4.15	2.50	1.29
(%)	G2	75.74	4.61	74.95	5.03	1.58	1.21
	G3	72.68	5.99	74.29	3.73	3.25	3.62
O_2_Hb_max_	G1	77.89	17.22	73.76	15.67	7.45	6.08
(µM)	G2	82.13	11.77	80.38	10.05	3.02	1.90
	G3	81.07	12.89	84.40	11.69	5.10	4.37
HHb_max_	G1	50.67	6.78	51.13	9.05	7.37	4.62
(µM)	G2	64.32	10.78	61.95	8.87	3.18	2.30
	G3	63.43	9.09	66.45	9.82	4.99	3.87
tHb_max_	G1	128.42	23.01	124.81	23.05	7.42	5.39
(µM)	G2	119.27	17.95	116.62	15.37	3.17	1.78
	G3	118.53	16.76	123.22	17.74	5.06	4.55

Legend: TSI: tissue saturation index; O_2_Hb: oxyhemoglobin; HHb: deoxyhemoglobin, tHb: total hemoglobin; CV: coefficient of variation; SD: standard deviation; btw: between. CV mean and CV SD between trials are calculated for the same participant and averaged per group.

**Table 10:** Between-session reliability of minimums.

Variable	Condition	SEM	ICC [CI-95%]	F
TSI	G1_R1	3.15	0.64 [0.08, 0.90]	4.51
(%)	G1_R2	3.03	0.59 [0.02, 0.88]	5.07
	G1_R3	2.44	0.7 [0.21, 0.91]	6.36
	G2_R1	5.33	0.71 [0.33, 0.89]	5.72
	G2_R2	5.10	0.71 [0.33, 0.89]	5.64
	G2_R3	5.35	0.69 [0.30, 0.89]	5.29
	G3_R1	7.69	0.44 [−0.08, 0.77]	2.52
	G3_R2	7.73	0.39 [−0.15, 0.74]	2.22
	G3_R3	7.30	0.39 [−0.15, 0.75]	2.21
O_2_Hb	G1_R1	4.13	0.77 [0.35, 0.94]	8.13
(µM)	G1_R2	4.10	0.75 [0.29, 0.93]	8.56
	G1_R3	3.83	0.8 [0.38, 0.95]	8.40
	G2_R1	6.57	0.79 [0.41, 0.93]	10.95
	G2_R2	6.79	0.76 [0.42, 0.91]	7.01
	G2_R3	6.02	0.78 [0.48, 0.92]	8.39
	G3_R1	11.06	0.26 [−0.30, 0.68]	1.66
	G3_R2	10.05	0.24 [−0.32, 0.67]	1.60
	G3_R3	10.49	0.24 [−0.33, 0.67]	1.60
HHb	G1_R1	1.88	0.8 [0.40, 0.95]	8.77
(µM)	G1_R2	2.37	0.7 [0.17, 0.92]	5.39
	G1_R3	1.72	0.77 [0.35, 0.94]	8.13
	G2_R1	1.97	0.86 [0.65, 0.95] *	13.22
	G2_R2	1.71	0.89 [0.71, 0.96] *	16.41
	G2_R3	1.94	0.86 [0.63, 0.95] *	12.29
	G3_R1	3.14	0.76 [0.42, 0.91]	6.92
	G3_R2	3.06	0.78 [0.46, 0.92]	7.69
	G3_R3	2.91	0.8 [0.50, 0.93] *	8.59
tHb	G1_R1	5.47	0.79 [0.40, 0.94]	8.98
(µM)	G1_R2	5.45	0.79 [0.38, 0.94]	9.74
	G1_R3	4.95	0.81 [0.43, 0.95]	9.29
	G2_R1	9.56	0.7 [0.27, 0.89]	6.93
	G2_R2	8.92	0.7 [0.33, 0.89]	5.78
	G2_R3	8.78	0.73 [0.38, 0.90]	6.62
	G3_R1	12.78	0.48 [−0.01, 0.79]	2.87
	G3_R2	10.98	0.55 [0.07, 0.82]	3.34
	G3_R3	11.30	0.53 [0.05, 0.81]	3.20

Legend: G1: group 1; G2: group 2; G3: group 3; R1: first reperfusion; R2: second reperfusion; R3: third reperfusion. Standard error of measurement (SEM) is expressed in arbitrary units (u.a.). Intraclass correlation coefficient (ICC) type: (2, 1); df: 9, 14, 14 for G1, G2 and G3, respectively. *p* < 0.05 in all conditions. * Moderate reliability.

**Table 11:** Between-session reliability of maximums.

Variable	Condition	SEM	ICC [CI-95%]	F
TSI	G1_R1	2.56	0.7 [0.22, 0.92]	6.44
(%)	G1_R2	1.99	0.8 [0.41, 0.95]	9.09
	G1_R3	3.09	0.53 [−0.07, 0.85]	3.29
	G2_R1	2.51	0.78 [0.48, 0.92]	8.41
	G2_R2	2.14	0.76 [0.43, 0.91]	7.22
	G2_R3	1.98	0.82 [0.55, 0.93] *	10.08
	G3_R1	4.81	0.07 [−0.45, 0.55]	1.14
	G3_R2	4.96	0.05 [−0.46, 0.54]	1.11
	G3_R3	4.81	0.05 [−0.47, 0.54]	1.10
O_2_Hb	G1_R1	11.17	0.55 [−0.05, 0.86]	3.45
(µM)	G1_R2	10.37	0.58 [0.02, 0.88]	3.90
	G1_R3	10.59	0.57 [−0.08, 0.87]	3.44
	G2_R1	4.58	0.84 [0.59, 0.94] *	11.48
	G2_R2	4.09	0.85 [0.63, 0.95] *	13.26
	G2_R3	4.22	0.84 [0.60, 0.94] *	11.28
	G3_R1	8.25	0.53 [0.07, 0.81]	3.37
	G3_R2	8.24	0.54 [0.08, 0.82]	3.43
	G3_R3	8.17	0.58 [0.12, 0.83]	3.72
HHb	G1_R1	6.36	0.36 [−0.38, 0.80]	2.01
(µM)	G1_R2	6.00	0.42 [−0.28, 0.82]	2.34
	G1_R3	6.76	0.26 [−0.50, 0.75]	1.62
	G2_R1	4.31	0.81 [0.46, 0.93]	12.05
	G2_R2	3.43	0.87 [0.67, 0.95] *	15.48
	G2_R3	3.73	0.86 [0.64, 0.95] *	14.69
	G3_R1	6.57	0.58 [0.14, 0.83]	3.86
	G3_R2	5.80	0.61 [0.19, 0.85]	4.34
	G3_R3	5.73	0.6 [0.17, 0.84]	4.23
tHb	G1_R1	16.76	0.45 [−0.22, 0.83]	2.57
(µM)	G1_R2	15.97	0.49 [−0.18, 0.85]	2.79
	G1_R3	17.30	0.43 [−0.29, 0.83]	2.37
	G2_R1	6.95	0.83 [0.58, 0.94] *	11.88
	G2_R2	6.37	0.85 [0.62, 0.95] *	12.38
	G2_R3	6.18	0.85 [0.63, 0.95] *	12.70
	G3_R1	13.59	0.4 [−0.11, 0.75]	2.31
	G3_R2	12.62	0.45 [−0.04, 0.77]	2.67
	G3_R3	12.52	0.47 [−0.03, 0.78]	2.73

Legend: G1: group 1; G2: group 2; G3: group 3; R1: first reperfusion; R2: second reperfusion; R3: third reperfusion. Standard error of measurement (SEM) is expressed in arbitrary units (u.a.). Intraclass correlation coefficient (ICC) type: (2, 1); df: 9, 14, 14 for G1, G2 and G3, respectively. *p* < 0.05 in all conditions. * Moderate reliability.

**Table 12:** Between-session variability of reoxygenation rate.

Variable	Groupe	Mean_S1	SD_S1	Mean_S2	SD_S2	Mean of CV Btw. Sessions (%)	SD of CV Btw. Sessions (%)
TSI_Slope_	G1	0.45	0.22	0.62	0.35	28.65	35.03
(%·s^−1^)	G2	1.18	0.34	1.00	0.33	11.98	8.68
	G3	1.37	0.33	1.30	0.30	9.25	8.76
O_2_Hb_Slope_	G1	0.29	0.33	0.66	0.39	65.91	73.73
(µM·s^−1^)	G2	1.47	0.52	1.36	0.47	15.55	18.79
	G3	1.72	0.39	1.80	0.49	9.21	3.67
HHb_Slope_	G1	0.37	0.30	0.70	0.37	22.64	13.54
(µM·s^−1^)	G2	1.11	0.89	1.47	0.66	13.67	6.42
	G3	1.77	0.47	1.82	0.91	10.04	8.43
tHb_Slope_	G1	0.42	0.22	0.62	0.21	49.19	59.65
(µM·s^−1^)	G2	1.34	0.46	1.29	0.48	47.65	56.06
	G3	1.64	0.27	1.72	0.57	21.73	29.31
TSI_Vpeak_	G1	0.95	0.28	1.18	0.38	16.40	9.67
(%·s^−1^)	G2	2.25	0.62	2.27	0.68	12.33	10.76
	G3	2.79	0.42	2.91	0.80	10.20	6.51
O_2_Hb_Vpeak_	G1	3.17	2.13	2.51	1.98	17.47	18.02
(µM·s^−1^)	G2	2.74	0.94	2.54	0.86	12.13	10.81
	G3	2.87	0.87	3.04	0.82	6.76	2.94
HHb_Vpeak_	G1	2.21	0.96	2.02	0.96	14.05	10.13
(µM·s^−1^)	G2	1.88	0.58	1.65	0.54	11.60	7.67
	G3	1.98	0.61	2.07	0.49	7.32	3.97
tHb_Vpeak_	G1	5.31	3.08	4.47	2.89	15.07	15.56
(µM·s^−1^)	G2	2.41	0.84	2.40	0.74	16.23	9.26
	G3	2.42	0.71	2.51	0.52	10.40	3.95

Legend: TSI: tissue saturation index; O_2_Hb: oxyhemoglobin; HHb: deoxyhemoglobin, tHb: total hemoglobin; CV: coefficient of variation; SD: standard deviation; btw: between. CV mean and CV SD between trials are calculated for the same participant and averaged per group.

**Table 13:** Between sessions reliability for the slope of reoxygenation rate.

Variable	Condition	SEM	ICC [CI-95%]	F
TSI_Slope_	G1_R1	0.29	0.38 [−0.15, 0.80]	3.00
(%·s^−1^)	G1_R2	0.17	0.34 [−0.17, 0.78]	2.60
	G1_R3	0.14	0.45 [−0.12, 0.83]	3.18
	G2_R1	0.22	0.75 [0.40, 0.91]	7.06
	G2_R2	0.22	0.79 [0.45, 0.93]	7.86
	G2_R3	0.28	0.65 [0.21, 0.87]	4.59
	G3_R1	0.35	0.43 [−0.10, 0.78]	2.55
	G3_R2	0.37	0.43 [−0.17, 0.79]	2.39
	G3_R3	0.26	0.62 [0.12, 0.87]	4.06
O_2_Hb_Slope_	G1_R1	0.33	0.42 [−0.13, 0.82]	4.42
(µM·s^−1^)	G1_R2	0.36	0.31 [−0.19, 0.76]	2.35
	G1_R3	0.33	0.23 [−0.19, 0.70]	2.04
	G2_R1	0.31	0.7 [0.28, 0.89]	5.38
	G2_R2	0.25	0.74 [0.37, 0.91]	7.36
	G2_R3	0.27	0.72 [0.35, 0.90]	6.61
	G3_R1	0.24	0.7 [0.27, 0.90]	5.53
	G3_R2	0.33	0.56 [0.06, 0.84]	3.57
	G3_R3	0.25	0.69 [0.24, 0.89]	5.14
HHb_Slope_	G1_R1	0.20	0.6 [−0.01, 0.89]	5.56
(µM·s^−1^)	G1_R2	0.20	0.6 [0.00, 0.89]	5.23
	G1_R3	0.15	0.67 [0.11, 0.91]	5.94
	G2_R1	0.20	0.65 [0.02, 0.89]	8.09
	G2_R2	0.24	0.56 [0.09, 0.83]	4.35
	G2_R3	0.24	0.56 [0.10, 0.83]	3.78
	G3_R1	0.24	0.42 [−0.17, 0.78]	2.35
	G3_R2	0.21	0.63 [0.17, 0.87]	4.47
	G3_R3	0.19	0.74 [0.35, 0.91]	6.54
tHb_Slope_	G1_R1	0.31	0.4 [−0.14, 0.81]	3.47
(µM·s^−1^)	G1_R2	0.34	0.3 [−0.20, 0.75]	2.26
	G1_R3	0.28	0.29 [−0.16, 0.74]	2.52
	G2_R1	0.45	0.73 [0.35, 0.90]	7.02
	G2_R2	0.59	0.37 [−0.09, 0.73]	2.50
	G2_R3	0.58	0.52 [0.06, 0.81]	3.54
	G3_R1	0.42	0.75 [0.37, 0.92]	6.80
	G3_R2	0.69	0.31 [−0.29, 0.73]	1.87
	G3_R3	0.50	0.42 [−0.16, 0.78]	2.39

Legend: G1: group 1; G2: group 2; G3: group 3; R1: first reperfusion; R2: second reperfusion; R3: third reperfusion. Standard error of measurement (SEM) is expressed in arbitrary units (u.a.). Intraclass correlation coefficient (ICC) type: (2, 1); df: 8, 13, 12 for G1, G2 and G3, respectively. *p* < 0.05 in all conditions.

**Table 14:** Between sessions reliability for Vpeak of reoxygenation rate.

Variable	Condition	SEM	ICC [CI-95%]	F
TSI_Vpeak_	G1_R1	0.31	0.4 [−0.18, 0.81]	2.62
(%·s^−1^)	G1_R2	0.28	0.48 [−0.10, 0.84]	3.47
	G1_R3	0.23	0.43 [−0.13, 0.82]	3.43
	G2_R1	0.42	0.56 [0.04, 0.83]	3.35
	G2_R2	0.48	0.45 [−0.11, 0.79]	2.55
	G2_R3	0.50	0.45 [−0.12, 0.79]	2.52
	G3_R1	0.55	0.36 [−0.18, 0.74]	2.16
	G3_R2	0.53	0.46 [−0.13, 0.80]	2.57
	G3_R3	0.43	0.39 [−0.22, 0.77]	2.18
O_2_Hb_Vpeak_	G1_R1	1.61	0.46 [−0.20, 0.85]	2.74
(µM·s^−1^)	G1_R2	1.11	0.67 [0.12, 0.91]	5.37
	G1_R3	1.26	0.59 [−0.06, 0.89]	3.74
	G2_R1	0.42	0.77 [0.44, 0.92]	7.61
	G2_R2	0.64	0.46 [−0.04, 0.79]	2.76
	G2_R3	0.57	0.66 [0.25, 0.88]	5.00
	G3_R1	0.28	0.9 [0.71, 0.97] *	20.76
	G3_R2	0.39	0.8 [0.45, 0.93]	10.36
	G3_R3	0.33	0.87 [0.62, 0.96] *	13.14
HHb_Vpeak_	G1_R1	0.71	0.46 [−0.27, 0.85]	2.60
(µM·s^−1^)	G1_R2	0.50	0.69 [0.11, 0.92]	5.19
	G1_R3	0.63	0.56 [−0.13, 0.88]	3.38
	G2_R1	0.33	0.65 [0.19, 0.87]	5.89
	G2_R2	0.30	0.74 [0.38, 0.91]	7.24
	G2_R3	0.33	0.68 [0.26, 0.89]	6.42
	G3_R1	0.32	0.74 [0.34, 0.91]	6.31
	G3_R2	0.27	0.77 [0.43, 0.92]	8.36
	G3_R3	0.22	0.81 [0.49, 0.94]	9.06
tHb_Vpeak_	G1_R1	2.31	0.46 [−0.23, 0.84]	2.64
(µM·s^−1^)	G1_R2	1.58	0.68 [0.14, 0.92]	5.42
	G1_R3	1.86	0.59 [−0.07, 0.89]	3.70
	G2_R1	0.71	0.31 [−0.26, 0.72]	1.87
	G2_R2	0.71	0.14 [−0.44, 0.62]	1.31
	G2_R3	0.55	0.7 [0.28, 0.89]	5.37
	G3_R1	0.32	0.75 [0.35, 0.92]	6.49
	G3_R2	0.50	0.57 [0.10, 0.84]	4.11
	G3_R3	0.35	0.78 [0.42, 0.93]	7.63

Legend: G1: group 1; G2: group 2; G3: group 3; R1: first reperfusion; R2: second reperfusion; R3: third reperfusion. Standard error of measurement (SEM) is expressed in arbitrary units (u.a.). Intraclass correlation coefficient (ICC) type: (2, 1); df: 8, 13, 12 for G1, G2 and G3, respectively. *p* < 0.001 in all conditions. * Moderate reliability.

## Data Availability

The data presented in this study are available on request from the corresponding author. The data are not publicly available due to ethical restrictions.
